# Association between ambient temperature and stroke risk in high-risk populations: a systematic review

**DOI:** 10.3389/fneur.2023.1323224

**Published:** 2024-01-08

**Authors:** Nathan Danh, Chau Ho, Emily Ford, Jian Zhang, Hua Hong, Christopher Reid, Dan Xu

**Affiliations:** ^1^Curtin Medical School, Faculty of Health Sciences, Curtin University, Perth, WA, Australia; ^2^Curtin School of Population Health, Faculty of Health Sciences, Curtin University, Perth, WA, Australia; ^3^Department of Neurology, First Affiliated Hospital, Sun Yat-sen University, Guangzhou, China

**Keywords:** ambient temperature, high cardiovascular risk population, stroke risk, stroke morbidity, stroke mortality, ischaemic stroke, haemorrhagic stroke

## Abstract

**Background:**

Significant associations exist between ambient temperature and stroke risk, but results in high cardiovascular risk populations are lacking. This systemic review summarised current evidence on ambient temperature and overall stroke risk in a high cardiovascular risk population.

**Methods:**

We performed a systematic literature search across MEDLINE, Embase, PsycINFO, CINAHL, Web of Science, and GEOBASE, from inception to 3 July 2023, to identify all population-based studies. Eligible studies screened by independent reviewers recruited individuals aged 18 years and over, where minimum 80% of participants had a high cerebral vascular disease (CVD) risk profile. The primary outcomes are stroke morbidity and mortality, while the secondary outcomes are morbidity and mortality of ischaemic stroke (IS), intracranial cerebral haemorrhage (ICH), and subarachnoid haemorrhage (SH).

**Results:**

The database searches identified 9,025 articles. After removing duplicates, 7,647 articles were screened in title and abstract to identify 380 articles for full-text screening. After the full-text screening of 380 articles by two independent reviewers, 23 articles were included in the review.

**Conclusion:**

The evidence for an association between ambient temperature and stroke incidence is that lower temperatures were more likely to increase morbidity and mortality risk of both haemorrhagic and ischaemic stroke in older people. Conversely, higher ambient temperature is significantly associated with intracranial haemorrhage risk, but decreased risk with IS. Higher and lower ambient temperatures consistently increase stroke risks in patients with comorbidities of congestive heart failure and dyslipidaemia. This evidence implies the need to establish clinical guidelines for preventive intervention in patients with high stroke risks during extreme ambient temperatures.

## Highlights

- Lower ambient temperatures are more likely to increase the morbidity and mortality risk of both ICH and IS in the elderly.
- Higher ambient temperatures are significantly associated with intracranial haemorrhage risk, but decreased risk of ischaemic stroke.
- Higher and lower ambient temperatures consistently increase stroke risks in patients with comorbidities of congestive heart failure and dyslipidaemia.
- Increased stroke risk was not observed, as compared to summer, in spring, autumn, or winter in stroke patients with comorbidities, including hypertension, diabetes, drinking, or smoking.
- Higher and lower ambient temperatures should be considered important influential factors when establishing clinical guidelines for preventive intervention in patients with high stroke risks.

## Background

Stroke accounts for 6.5 million deaths each year and is the world’s second leading cause of mortality (1). Identification of modifiable risk factors for stroke is essential to mortality reduction. While risk factors, such as hypertension and diabetes, are well established, environmental risk factors for stroke remain to be investigated (1, 2). Acute stroke is not distributed randomly over time but depends on months/seasons of the year. Cold temperature was associated with an increased incidence of stroke morbidity and mortality (3–9) that may be attributable to the effect of cold temperature on increased blood pressure and serum cholesterol (10, 11). Hot temperature was associated with an increased risk of ischaemic stroke (IS) mortality but a decreased risk of intracerebral haemorrhage (ICH) mortality (12). A study by Salam et al. reported an increased incidence of IS relative to ICH during the summer months with higher solar radiation that cannot be explained by physiological measures suggestive of dehydration or hem-concentration (13). However, in a study by Cowperthwaite et al. (14), no significant association between weather and stroke incidence was recorded in the USA. A lack of evidence was reported in people with a high CVD risk profile, such as those with old age or CVD risk factors (e.g., a history of CVD, hypertension, and diabetes). Thus, in this systematic review, we aimed to investigate the association between extreme temperature and the relative risks of stroke morbidity and mortality in a high CVD risk population to inform the guidelines for the development of stroke preventive interventions.

## Methods

### Criteria for considering studies

Types of studies: All types of studies with a minimum 1-year duration to ensure all seasons were presented.

Types of participants: This review included individuals aged 18 years and over, where at least 80% of participants who had a high CVD risk profile, including the elderly who were 65 years or over, diabetes (defined as a previous diagnosis of type 1 or type 2 diabetes and/or current treatment with therapies to lower blood glucose levels), previous history of CVD (defined as myocardial infarction, angina pectoris, coronary bypass surgery, coronary angioplasty, stroke, transient ischaemic attack, carotid endarterectomy, surgery for peripheral vascular disease, intermittent claudication or renal failure determined as creatinine >1.5 times the upper limit of normal or chronic kidney diseases determined as eGFR<30 mL/min/1.73 m^2^), high blood pressure (defined as previous diagnosis of high blood pressure or on treatment with BP lowering drugs), and high blood lipid (defined as a previous diagnosis of high blood lipid or on treatment with lipid-lowering drugs).

Types of exposure: This review focused on ambient temperature, which is defined as the temperature of the surrounding air. As previous studies have shown the relatively same predictive ability of different temperature measures for health outcomes (15), we examined all available measurements of ambient temperature, such as daily or monthly mean, maximum or minimum temperatures, temperature change, or variation.

### Type of outcome measures

Primary outcomes: Stroke morbidity and mortality.

Secondary outcomes: IS morbidity and mortality, ICH morbidity and mortality, subarachnoid haemorrhage (SH) morbidity and mortality.

Language: No restriction (English and non-English studies).

Publication type: Published and unpublished studies reported in peer-reviewed journals, reports, conference abstracts, and theses.

### Search methods for identification of studies

We performed a similar search strategy developed by Wang et al. (16) in Ovid Medline, Ovid Embase, CINHL, Web of Science, and PsycINFO (Ovid). We combined the search terms relating to ‘ambient temperature’ and ‘stroke’ based on the strategy used by Wang et al. (16). We also searched reference lists of known previous systematic reviews and meta-analyses of the association between ambient temperature and stroke (17–19). Corresponding authors of relevant studies were contacted regarding any further published or unpublished study if needed.

### Study selection

First, two independent reviewers screened a small sample of papers found in the search to revise any unclear or inappropriate inclusion criteria. In the full selection process, two reviewers independently scanned the results of the search and determined the eligibility of the studies. In the initial screening of titles and abstracts, the studies were included if they met the inclusion criteria or if they did not have enough information for exclusion. Rejected citations were recorded and classified as irrelevant studies. All potentially relevant articles were screened in full text for a final decision. If a study did not have sufficient information to assess eligibility, we attempted to contact the authors; the study was classified as a potentially relevant article and checked in sensitivity analyses if authors did reply after 1 month.

### Data extraction and quality assessment

Data extraction forms and quality assessment forms were piloted on a small group of studies. Two reviewers independently performed data extraction and quality assessment in the prespecified form. If any disagreements were raised, the reviewers would discuss consensus or consult with the third reviewer. A report of corrections or amendments to the prespecified form would be recorded.

### Quality assessment

The quality of the included studies was assessed following the Strengthening the Reporting of Observational Studies in Epidemiology (STROBE) statement (20). The quality criteria included setting, locations, and dates for the recruitment period; eligibility criteria, sources, and methods of selecting participants; consecutive or random participant sampling; the proportion of eligible participants (participant rate); the number of participants in each stage of the study or reasons for ineligibility; a description of the included participants; a description of ambient temperature measurement and stroke; and sources of funding acknowledgement. The bias was assessed as low risk (sufficient data were provided and fulfilled the criteria), partial risk (insufficient data were provided), or high risk (sufficient data were provided and did not fulfil the criteria). Publication bias was judged by observing the asymmetry of funnel plots; if they were asymmetric, contour-enhanced funnel plots were then analysed to examine whether publication bias alone caused the asymmetry (21).

Due to the substantial inconsistencies of the measurement for exposure (e.g., ambient temperature), we reviewed and summarised findings from included studies and did not process with a meta-analysis.

### Ethics and dissemination

This systematic review analysed non-identifiable data; thus, a formal ethics approval is unlikely to be crucial.

## Results

### Result of searches

**Figure 1:**
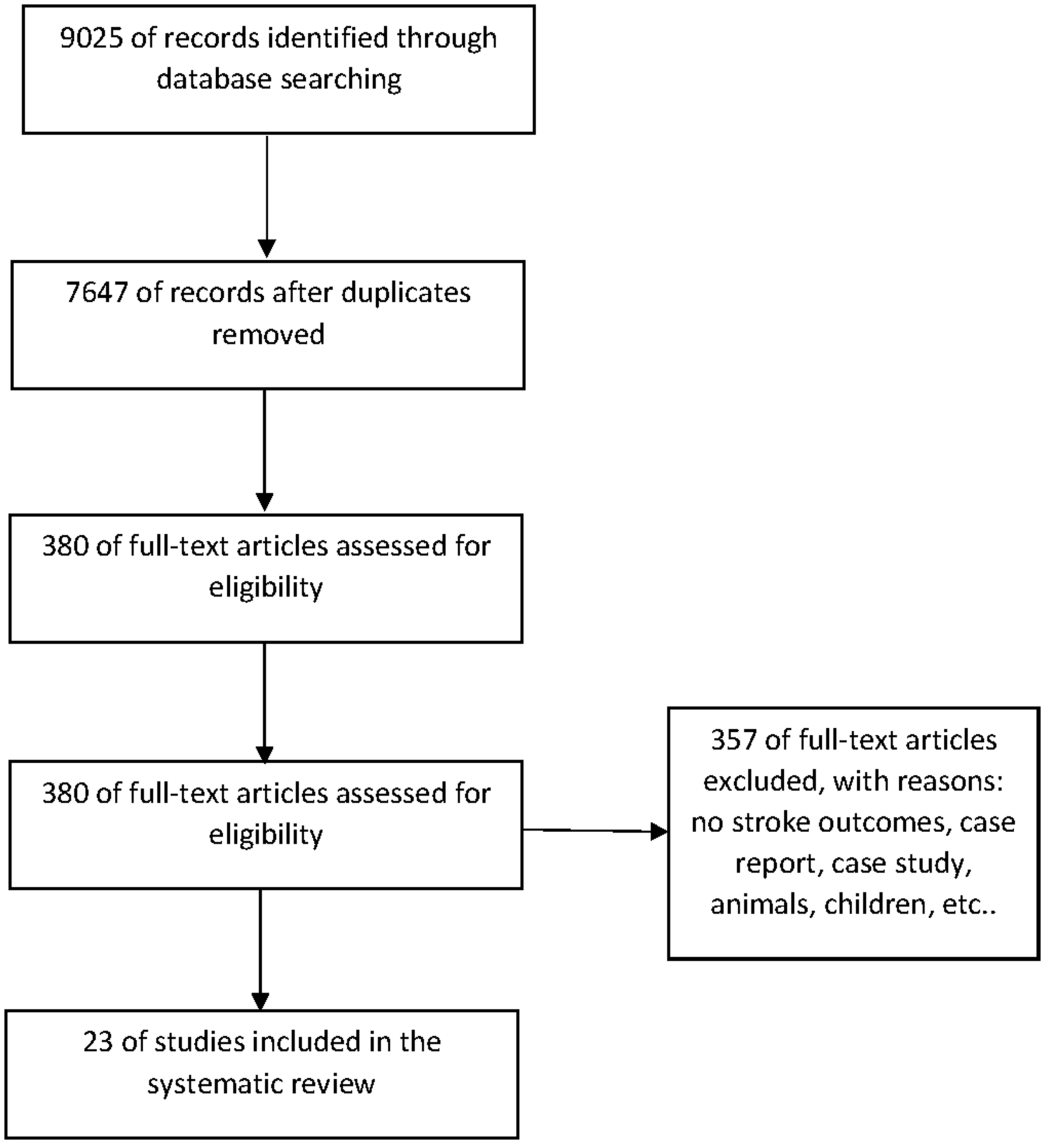
Flow diagram of eligible articles for the review.

As presented in the flow chart (Figure 1), the database searches identified 9,025 articles. After removing duplicates, 7,647 articles were screened. A total of 380 articles were screened in full-text, with 65 potentially appropriate studies for full-text screening to be included in the systematic review. A total of 23 articles were included in the final systematic review. Most of the articles were excluded due to a lack of a high CVD risk cohort, stroke outcomes, exposure to ambient temperature, or unclear results.

### Characteristics of included studies

As presented in Table 1, most of the included studies were conducted in Asian countries, such as China and Japan, and were designed as time-series or time-stratified case–crossover study. However, a large number of studies did not sufficiently detail their study design, as mentioned in Table 1. More results were reported for the elderly group (>65 years) than those with CVD risk factors (e.g., hypertension, diabetes, and a history of CVD). Data on the high-risk population were substantially extracted from subgroup analyses, whereas two studies were restricted to the elderly. Diagnosis of stroke or stroke sub-type was based on the stratification of ICD-9th or 10th except for 10 studies (30, 31, 33–35, 40, 41, 43–45) that confirmed stroke by brain imaging, such as CT, MRI, or cerebral angiogram.

**Table 1:** Characteristics of included studies.

First author (year)	Study design	Study population	City, country, data collection time	Ambient temperature/seasonal variation	Stroke measurement
Bai et al. (2018) (22)	Case-crossover study	Ontario residents admitted to a hospital	Ontario, Canada, 1996–2013	Daily mean temp: −33.1°C to 32.2°C	Stroke hospitalisation based on ICD-9 and ICD-10.
Goggin et al. (2012) (23)	Time series study	Patients registered in stroke admission	Hong Kong, China, 1999–2006	Daily mean temp: 8.2°C to 31.8°C	Stroke hospitalisation based on ICD-9
Guo et al. (2017) (24)	Case-crossover study	Patients admitted to hospital with stroke	Guangzhou, China, 2013–2015	Daily mean temp: 4.8°C to 21.9°C	Stroke hospitalisation based on ICD-10
Hasegawa et al. (2015) (25)	Cross-sectional study	Residence in 47 prefectures aged 65–89 years	Nationwide, Japan, 2010	Daily mean temp: 15.5°C ± 2.3°C	Stroke mortality data from population census data, stroke data based on ICD-10
Lee et al. (2008) (26)	Case-crossover study	Patients admitted to the emergency department for stroke	Taiwan, 1998–2003	Daily mean temp: 16.2°C to 29.3°C	Stroke hospitalisation based on ICD-9
Lichtman et al. (2016) (27)	Cross-sectional study	Hospitalised patients	USA, 2009–2011	Daily mean temp: −25°F to 100°F Diurnal temperature variation: 5–45°F (smallest in winter)	Stroke hospitalisation based on ICD-9
Lim et al. (2017) (28)	Case-crossover study	Patients admitted to the emergency department for stroke	Nationwide, South Korea, 1–12/2011	Daily mean temp: −9.8°C to 29.2°C	Stroke hospitalisation based on ICD-10
Luo et al. (2018) (6)	Retrospective time-series study	Patients from 63 hospitals with stroke	Beijing, China, 2013–2014	Daily mean temp: −12.9°C to 30.1°C	Stroke hospitalisation based on ICD-10
Pan et al. (1995) (29)	Unclear	Residents in selected areas	Nationwide, Taiwan, 1981–1991	NI	Stroke mortality based on ICD-8
Passero et al. (2000) (30)	Unclear	Patients admitted to neurological, neurosurgical, and intensive care departments of a university hospital	Siena, Italy, 1979–1996	Monthly mean temperature: 3°C to 26.1°C	Intracerebral haemorrhage from CT scan or autopsy.
Qi et al. (2020) (31)	Unclear	Patients admitted to the stroke unit of a hospital	Tianjin, China, 2014–2019	Daily mean temperature: 7.2 (3.1–11.6°C)	Stroke ischemic diagnosis based on MRI or CT
Ravljen et al. (2021) (32)	Time-series study	Patients admitted to a university medical centre for stroke	Ljubljana, Slovenia, 2012–2017	NI	Ischemic stroke based on ICD-10
Shinkawa et al. (1990) (33)	A retrospective cohort study (FU: 24 years)	Hisayama residents	Hisayama, Japan, 1961–1985	Monthly ambient temperature: 6–27°C	Stroke diagnosis based on histories, neurologic examination, lumbar puncture, cerebral angiography, CT, and autopsy.
Toyoda et al. (2018) (34)	Unclear	Patients admitted to the emergency department of the national cerebral and cardiovascular centre	Osaka, Japan, 2011–2015	NI	Ischemic stroke diagnosis based on neurological and radiological examinations (MRI, CT)
Turin et al. (2008) (35)	Unclear	Patients hospitalised with stroke in the county hospitals	Takashima, Japan, 1988–2001	Average annual temperature: 13.5 (2.5–29.5°C)	Stroke diagnosis based on clinical symptoms, CT or MRI
Vered et al. (2020) (36)	Time-stratified case-crossover study	Patients admitted to 26 general hospitals in summer	Nationwide, Israel, 2014–2016	Mean ambient temperature: 26.2 (21.6–31.4°C)	Stroke diagnosis based on ICD-9
Vodonos et al. (2017) (37)	Time-stratified case-crossover study	Patients admitted to a university medical centre	Southern Negev, Israel, 2012–2014	Daily ambient temperature: 19.1 (2.1–38.8)°C	Stroke diagnosis by neurologist
Wang et al. (2009) (38)	Time-stratified case-crossover study	Patients admitted to hospitals	Brisbane, Australia, 1996–2005	Mean daily temperature: 14.7–26.3°C	Stroke diagnosis based on ICD-9 and ICD-10
Yang et al. (2016) (7)	Time-stratified case-crossover study	Residents from 16 large Chinese cities	Nationwide, China, 2007–2013	Daily mean temperature: 5.1 to 21.6°C	Stroke deaths diagnosis from the China Information System of Death Register and China CDC
Zheng et al. (2016) (39)	A time-series study	Patients ≥65 years admitted to three comprehensive hospitals	Beijing, China, 2009–2011	Mean daily temperature: 13.15 (−12.5 to 34.5°C)	Stroke diagnosis based on ICD-10
Chang et al. (2004) (40)	Case control study	Female patients admitted to 160 hospitals	17 countries, 1989–1995	Mean monthly temperature: −4.6°C (Beijing) to 30.1°C (Thailand)	Stroke diagnosis based on clinical signs, CT, MRI, cerebral angiogram.
Palm et al. (2013) (41)	Time-stratified case-crossover study	Patients admitted to the hospital with stroke	Ludwigshafe, Germany, 2006–2010	NI	Stroke diagnosis based on WHO definition and brain imaging
Ikefuti et al. (2018) (42)	Time-series study	Resident in São Paulo with stroke	São Paulo, Brazil, 2002–2011	Mean daily temperature: 19.45°C (8.4–27.6°C)	Stroke mortality diagnosis based on ICD-10

ICD-8, International Classification of Disease Eighth Revision; ICD-9, International Classification of Disease Ninth Revision; ICD-10, International Classification of Disease 10th Revision; CT, computer tomography; MRI, magnetic resonance imaging; WHO, World Health Organisation; CDC, Centre of Disease Control and Prevention.

### Risk of bias

Nine out of the 23 included studies were assessed as having a high risk of bias due to a lack of information on eligibility criteria, characteristics of study participants, and source of funding. All of the studies provided sufficient definitions for stroke assessment. Most of the studies utilised admission data or stroke registry data, so consecutive samples were recruited. More information is provided in Table 2. As studies reported results in different comparisons, we were unable to assess publication bias.

**Table 2:** Risk of bias.

First author (year)	Setting, locations, and dates for recruitment provided	Eligibility criteria, sources, and methods of selecting participants provided	Consecutive or random participant sampling	Proportion of eligible participants (participants rate) described	Number of participants at each stage of study/reasons for ineligibility provided	Clear description of study participants	Clear description of stroke assessment	Source of funding acknowledged	Risk (Low/Partial/High)
Bai et al. (2018) (22)	Y	Y	Y	Y (100%)	N	Y	Y	Y	Low
Goggin et al. (2012) (23)	Y	Y	Y	Y (100%)	N	N	Y	Y	Low
Guo et al. (2017) (24)	Y	N	Y	Y (100%)	N	Y	Y	N	Low to partial
Hasegawa et al. (2015) (25)	Y	N	Y	Y (100%)	N	N	Y	N	Low to partial
Lee et al. (2008) (26)	Y	Y	Y	Y (100%)	N	N	Y	N	Low to partial
Lichtman et al. (2016) (27)	Y	Y	Y	Y (1.7%)	N	Y	Y	N	Low
Lim et al. (2017) (28)	Y	Y	Y	N	Y	N	Y	Y	Low
Luo et al. (2018) (6)	Y	N	N	N	N	N	Y	Y	High
Pan et al. (1995) (29)	Y	N	N	N	N	N	Y	N	High
Passero et al. (2000) (30)	Y	Y	Y	Y (100%)	Y	Y	Y	Y	Low
Qi et al. (2020) (31)	Y	Y	N	N	N	Y	Y	Y	Low
Ravljen et al. (2021) (32)	Y	N	N	N	N	N	Y	Y	High
Shinkawa et al. (1990) (33)	Y	Y	N	N	Y	Y	Y	Y	Low
Toyoda et al. (2018) (34)	Y	Y	Y	N	N	Y	Y	Y	Low
Turin et al. (2008) (35, 46)	Y	Y	Y	Y (100%)	N	Y	Y	Y	Low
Vered et al. (2020) (36)	Y	Y	Y	Y (100%)	Y	Y	Y	Y	Low
Vodonos et al. (2017) (37)	Y	Y	Y	Y (100%)	Y	Y	Y	N	Low
Wang et al. (2009) (38)	Y	N	N	N	N	N	Y	Y	High
Yang et al. (2016) (7)	Y	N	N	N	N	N	Y	Y	High
Zheng et al. (2016) (39)	Y	Y	N	N	N	N	Y	N	High
Chang et al. (2004) (40)	Y	Y	N	Y (100%)	N	N	Y	Y	Low to partial
Palm et al. (2013) (41)	Y	Y	Y	Y (100%)	N	Y	Y	Y	Low
Ikefuti et al. (2018) (42)	Y	N	N	N	N	N	Y	Y	High

### Association between ambient temperature and stroke in the elderly

As shown in Table 3A, most of the included studies showed a significant association between ambient temperature or seasonal pattern and stroke in the elderly, except for the studies by Luo et al. (6) (Table 3B), Vodonos et al. (37) (Table 3B), and Lee et al. (26) (Table 3B). Exposure to cold or lower temperature was more likely to increase the morbidity and mortality risk of stroke and stroke sub-type by a magnitude of 10–50% (22, 23, 29, 30) (Table 3A). An increase in mean ambient temperature was associated with a decreased risk of morbidity of general stroke and haemorrhagic stroke by a magnitude of 1–7% (27, 38). Studies by Zheng et al. (39) (Table 3A), Qi et al. (31) (Table 3A), and Lim et al. (28) (Table 3A) showed a positive association between a 1°C increase in diurnal temperature range and the risk of general stroke and ischaemic stroke morbidity that varied from 1.5 to 3.1%. In contrast to these observations, Vered et al. (36) (Table 3A) reported that a 1°C increase in a diurnal temperature range was associated with a reduced risk of general stroke and stroke sub-type morbidity by 1–3% and a 1°C increase in mean ambient temperature was associated with an increased risk of general stroke and stroke sub-type morbidity by a magnitude of 7–10%.

**Table 3:** Association between ambient temperature and stroke in the elderly.

A. Significant results								
First author (year)	Exposure/lag days	Stroke morbidity	Stroke mortality	Haemorrhage stroke	Haemorrhage stroke mortality	Ischaemic stroke	Ischaemic stroke mortality	Variables controlled for
Hasegawa et al. (2015) (25)	“ambient temperature”/unavailable lag days	**Men, 65–69 years: Rho = −0.424, *p* < 0.01.** **Men, 85–89 years: −0.393, *p* < 0.01.** Women, 65–69 years: −0.275, *p* > 0.05 **Women, 85–89 years: −0.426, *p* < 0.01.**	**65–69 years: *β* = −0.478, *p* = 0.001.**	–	–	–	–	Salt intake, BMI, pedometer count, altitude, medical cost, yearly income, GiNi coefficient, and the number of nursing homes
Bai et al. (2018) (22)	−16 vs. 0.6°C/ 21 days	**↑RR 1.1 (95% CI 1.02–1.19)**	–	–	–	–	–	Relative humidity, NO_2_, O_3_, a day-of-week indicator, holiday status, daily influenza visits, seasonality.
	25.2 vs. 17°C/ 21 days	RR 1.033 (0.94–1.16)	–	–	–	–	–	
Goggin et al. (2012) (23)	15.2°C vs. 29.5°C/ 0–4 days	–	–	**↑ RR 1.52 (95%CI 1.38–1.67)**	–	–	–	Humidity, NO_2_, SO_2_, RSP, O_3_, a day-of-week indicator, holiday status, daily influenza visits, seasonality.
	15.7°C vs. 22°C/ 0–13 days	–	–	–	–	**↑ RR 1.15 (95%CI 1.11–1.19)**	**–**	
Pan et al. (1995) (29)	9 vs. 28°C/unavailable lag days	–	–	–	**↑ OR 1.42 (95%CI 1.11–1.80)**	–	–	–
	9 vs. 29°C/ unavailable lag days	–	–	–	–	–	**↑ OR 1.92 (1.50–2.46)**	
Passero et al. (2000) (30)	<6°C/unavailable lag days	–	–	**↑ RR = 1.25 (*p* < 0.0035) for those aged 60+ years**	–	–	–	–
Guo et al. (2017) (24)	9.47°C vs. 23.4°C 0–21 days	–	–	**65–74 year: f-AF**_**tot**_**11.3 (95% eCI -11.4–23.0)** **75+ year: f-AF**_**tot**_**24.0 (95% eCI 13.0–29.8)**	–	**65–74 year: f-AF**_**tot**_**8.5 (95% eCI -0.5–15.1)** **75+ year: f-AF**_**tot**_**11.2 (95% eCI 3.6–16.6)**	–	Day of the week, public holidays, long-term and seasonal trends of daily strokes, air pollution, and meteorological variables.
Lichtman et al. (2016) (27)	5°F ↑ in average temperature/ unavailable lag days	**↓ OR 0.97 (95%CI 0.96–0.97)**	–	–	–	–	–	Patient age, sex, race, dew point temperature, and the following comorbid conditions and medical history variables
Ravljen et al. (2021) (32)	1°C change in ambient temperature/ Lag 7	–	–	–	–	**↓ *β* = −0.006, *p* = 0.011**	–	–
Wang et al. (2009) (38)	1°C ↑ in minimum temperature in winter/ lag 3 months	–	–	**↓ RR 0.94 (0.90–0.98)**	–	RR 0.99 (0.97–1.01)	–	Humidity, PM_10_, NO_2_, O_3_, SO_2_
	1°C ↑ in minimum temperature in summer/ lag 3 months	–	–	RR 0.99 (0.94–1.05)	–	RR 1.00 (0.97–1.02)	–	
	1°C ↑ in maximum temperature in winter/ lag 3 months	–	–	**↓ RR 0.93 (0.89–0.96)**	–	RR 0.97 (0.94–1.00)	–	
	1°C ↑ in maximum temperature in summer/ lag 3 months	–	–	**↓ RR 0.93 (0.88–0.99)**		RR 0.99 (0.96–1.02)	–	
Vered et al. (2020) (36)	1°C ↑ in mean ambient temperature/ 0–6 days	**↑ OR 1.10 (1.08–1.13) for those 75+ years**	–	OR 1.07 (0.99–1.15) for those 75+ years	–	**↑ OR 1.11 (1.08–1.13) for those 75+ years**	–	Relative humidity and air pollution
	1°C ↑ in diurnal temperature range/0–6 days	**↓ OR 0.97 (0.95–0.98) for those 75+ years**	–	OR 0.99 (0.93–1.05) for those 75+ years	–	**↓ OR 0.97 (0.95–0.98) for those 75+ years**	–	
Zheng et al. (2016) (39)	1°C ↑ in diurnal temperature range/0–4 days	**↑ percentage of ER admission 2.13% (0.51–3.77) for men** **1.55% (0.19–2.93) for women**	–	–	–	–	–	Time trend, day of the week, holiday, temperature, relative humidity, wind speed, atmospheric pressure, and air pollutants.
Qi et al. (2020) (31)	1°C ↑ in diurnal variation/ 0–4 days	–	–	–	–	**↑ Daily number of ischemic stroke onset: 0.016 (0.005–0.028) for those aged 75+ years**	–	Day of week, public holiday, and air pollutants.
Lim et al. (2017) (28)	1°C change in diurnal temperature/unavailable lag days. Temperature changes over preceding 24 h/ unavailable lag days.	**↑ RR 1.027 (1.008–1.047)** **↓ RR 0.953 (0.921–0.985)**	– –	RR 0.982 (95%CI 0.91701.052) RR 0.9837 (95%CI 0.916–1.056)	– –	**↑ RR 1.031 (95%CI 1.011–1.052)** **↑ RR 0.949 (95%CI 0.916–0.983)**	– –	Diurnal temperature changes, diurnal variation of atmospheric pressure, PET, temperature differences over the previous day, and diurnal temperature change ≥10°C
Yang et al. (2016) (7)	Cold effect/0–14 days	–	**AF 14.9 (10.2–18.2) for those 65–74 years** **AF 14.0 (10.4–16.7) for those 75+ years**	**–**	–	–	–	–
	Heat effect/unavailable lag days	–	**AF 1.3 (0.9–1.6) for those 65–74 years** **AF 1.6 (1.3–2.0) for those 75+ years**	–	–	–	–	–
Turin et al. (2008) (35)	Spring vs. summer/unavailable lag days	**↑ OR 1.27 (1.09–1.48)**	–	–	–	–	–	–
	Autumn vs. summer/unavailable lag days	Non-significant	–	–	–	–	–	–
	Winter vs. summer/unavailable lag days	Non-significant	–	–	–	–	–	–
Shinkawa et al. (1990) (33)	Seasonal variation/unavailable lag days	Roger’s *R* = 1.18, *p* > 0.05 for those 65–74 years **+ Roger’s *R* = 7.03, *p* < 0.05 for those 75+ years**	–	Roger’s *R* = 5.32, *p* > 0.05 for those 65–74 years Roger’s *R* = 5.32, *p* > 0.05 for those 75+ years	–	Roger’s *R* = 4.07, *p* > 0.05 for those 65–74 years Roger’s *R* = 4.07, *p* > 0.05 for those 75+ years	–	–
Toyoda et al. (2018) (34)	Seasonal variation/unavailable lag days	**↑ incidence rate in winter and spring (*p* = 0.026) in those at 75+ years**	–	–	–	–	–	–

B. Non-significant results								
First author (year)	Exposure/lag days	Stroke morbidity	Stroke mortality	Haemorrhage stroke	Haemorrhage stroke mortality	Ischaemic stroke	Ischaemic stroke mortality	Variables controlled for
Luo et al. (2018) (6)	−9.6 vs. 1.2°C (extreme cold)/0–7 days for ischemic stroke, 0–3 days for haemorrhage stroke	–	–	RR 1.28 (95%CI 0.94–1.74)	–	RR 1.28 (95%CI 0.99–1.64)	–	Air pollution (PM2.5), day of the week, holiday.
	−4.2 vs. 1.2°C (moderate cold) / 0–7 days for ischemic stroke, 0–3 days for haemorrhage stroke	–	–	RR 1.07 (95%CI 0.97–1.19)	–	RR 1.06 (95%CI 0.98–1.15)	–	
	25.7 vs. 22.1°C (moderate hot) / 0–7 days for ischemic stroke, 0–3 days for haemorrhage stroke	–	–	RR 0.97 (95%CI 0.84–1.11)	–	RR 1.02 (95%CI 0.92–1.12)	–	
	28.4 vs. 22.1°C (extreme hot) / 0–7 days for ischemic stroke, 0–3 days for haemorrhage stroke	–	–	RR 0.96 (95%CI 0.77–1.20)	–	RR1.03 (95%CI 0.88–1.20)	–	
Vodonos et al. (2017) (37)	5°C ↑ in ambient temperature/0–4 days	–	–	–	–	OR 1.38 (0.83–2.29) for those aged 65–74 years OR 1.01 (0.71–1.45) for those aged 75+ years	–	–
Lee et al. (2008) (26)	Ambient temperature/unavailable lag days	Monthly stroke incidence rate: 65–74 years: *β* = −0.526; NS. >74 years: *β* = −1.067, NS.	–	–	–	–	–	Time-trend and monthly effects

BMI, body mass index; NO_2_, nitrogen dioxide; O_3_, ozone (trioxygen); SO_2_, sulphur dioxide; RSP, respirable suspended particulates; RR, relative risk; OR, odds ratio; PM_10_, particulate matter < 10 μm in diameter; PM_2.5_, particulate matter < 2.5 μm in diameter; ER, emergency room; PET, physiologically equivalent temperature; AF_tot_, total attributable fraction as computed forward; AF, atrial fibrillation; Roger’s R, Roger test for significance of the seasonal variation; β, beta coefficient; Bold, statistically significant.

### Association between ambient temperature and stroke in patients with comorbidities

As shown in Table 4, Vered et al. (36) reported a significant association between a 1°C increase in mean ambient temperature and general stroke and ischemic stroke in subgroups with hypertension, diabetes, atrial fibrillation, or hyperlipidaemia, whereas Vodonos et al. (Table 4 non-significant results) (37) and Bai et al. (22) did not show any significant effect of exposure to a 5°C increase in ambient temperature or hot effect on stroke or stroke sub-type in this population. However, Bai et al. (22) observed a significant effect of exposure to both cold (−16°C vs. −0.6°C) and hot temperatures (25.2°C vs. 17°C) on the increased risk of general stroke in those with congestive heart failure, with a RR of 1.12 (95%CI 1.89–1.41). Shinkawa et al. (33) reported a seasonal pattern of general stroke in patients with hypertension and ischemic stroke in patients with high total cholesterol. In the study (33), by Turin et al. (Table 4 non-significant results), as compared to summer, no significant difference in the risk of general stroke was recorded in spring, autumn, or winter in patients with hypertension, diabetes, drinking, or smoking.

**Table 4:** Association between ambient temperature and stroke in patients with comorbidities.

A. Significant results								
First author (year)	Exposure/lag days	Stroke morbidity	Stroke mortality	Haemorrhage stroke	Haemorrhage stroke mortality	Ischaemic stroke	Ischaemic stroke mortality	Variables controlled for
Bai et al. (2018) (22)	−16 vs. −0.6°C/21 days	**↑ RR 1.12 (1.89–1.41) for those with CHF** RR 1.13 (0.99–1.29) for those with HTN RR 0.94 (0.78–1.14) for those with DM RR 1.0 (0.74–1.35) for those with acute MI RR 1.19 (0.97–1.45) for those with cardiac dysrhythmias	–	–	–	–	–	Relative humidity, NO2, O3, a day-of-week indicator, holiday status, daily influenza visits, seasonality.
	25.2 vs. 17°C/21 days	**↑ RR 1.12 (1.89–1.41) for those with CHF** RR 1.13 (0.99–1.29) for those with HTN RR 0.96 (0.76–1.21) for those with DM RR 0.87 (0.62–1.19) for those with acute MI RR 1.19 (0.97–1.45) for those with cardiac dysrhythmias	–	–	–	–	–	
Vered et al. (2020) (36)	1°C increase in mean ambient temperature/0–6 days	**↑ OR 1.09 (1.08–1.11) for those with HTN** **↑ OR 1.09 (1.07–1.12) for those with DM** **↑ OR 1.08 (1.03–1.14) for those with AF** **↑ OR 1.10 (1.08–1.12) for those with hyperlipidaemia**	–	**↑ OR 1.08 (1.01–1.16) for those with HTN** OR 1.01 (0.92–1.10) for those with DM OR 1.19 (0.93–1.51) for those with AF OR 1.09 (0.99–1.20) for those with hyperlipidaemia	–	**↑ OR 1.10 (1.08–1.11) for those with HTN** **↑ OR 1.10 (1.08–1.13) for those with DM** **↑ OR 1.07 (1.02–1.13) for those with AF** **↑ OR 1.10 (1.08–1.12) for those with hyperlipidaemia**	–	Relative humidity and air pollution
	1°C increase in diurnal temperature range/0–6 days	**↓ OR 0.97 (0.95–0.98) for those with HTN** **↓ OR 0.96 (0.95–0.98) for those with DM** **↓ OR 0.97 (0.96–0.99) for those with hyperlipidaemia** OR 0.99 (0.95–1.03) for those with AF	–	OR 0.96 (0.91–1.02) for those with HTN OR 1.03 (0.95–1.12) for those with DM OR 0.94 (0.79–1.11) for those with AF OR 0.99 (0.91–1.07) for those with hyperlipidaemia	–	**↓ OR 0.97 (0.95–0.98) for those with HTN** **↓ OR 0.96 (0.94–0.98) for those with DM** **↓ OR 0.97 (0.96–0.99) for those with hyperlipidaemia** OR 0.99 (0.95–1.03) for those with AF	–	
Shinkawa et al. (1990) (33)	Seasonal variation/unavailable lag days	**Roger’s *R* = 10.44, *p* < 0.01 for those with HTN** ± Roger’s *R* = 3.1, *p* > 0.05 for those with high TC	–	**Roger’s *R* = 8.57, *p* < 0.05 for those with HTN** **Roger’s *R* = 8.96, *p* < 0.05 for those with high TC**	–	Roger’s *R* = 5.07, *p* > 0.05 for those with HTN Roger’s *R* = 1.41, *p* > 0.05 for those with high TC	–	–

B. Non-significant results								
First author (year)	Exposure/lag days	Stroke morbidity	Stroke mortality	Haemorrhage stroke	Haemorrhage stroke mortality	Ischaemic stroke	Ischaemic stroke mortality	Variables controlled for
Vodonos et al. (2017) (37)	5°C increase in ambient temperature/0–4 days	–	–	–	–	OR 1.07 (0.73–1.56) for those with HTN OR 1.11 (0.82–1.50) for those with DM OR 1.04 (0.62–1.76) for those with AF OR 1.16 (0.81–1.66) for those with dyslipidaemia	–	–
Turin et al. (2008) (35)	Spring vs. summer Autumn vs. summer/winter vs. summer/ / unavailable lag days	Non-significant OR for those with HTN, DM, drinking or smoking	–	–	–	–	–	

RR, relative risk; OR, odds ratio; CHF, congestive heart failure; HTN, hypertension; DM, diabetes mellitus; MI, myocardial infarction; AF, atrial fibrillation; NO_2_, nitrogen dioxide; O_3_, ozone (trioxygen); Roger’s R, Roger test for significance of the seasonal variation; High TC, high total cholesterol. Bold, statistically significant.

## Discussion

In this systemic review, a consistent observation in the elderly indicated that lower temperature was more likely to increase the morbidity and mortality risk of both haemorrhagic and ischaemic strokes in a temperature-dependent manner with a magnitude of 10–50%. Increased ambient temperature was associated with decreased stroke morbidity, especially haemorrhagic stroke, in a temperature-dependent manner with a magnitude of 1–7%. An increase in the diurnal temperature range was associated with an elevated risk of stroke, in particular, ischemic stroke morbidity, which varied from 1.5 to 3.1%. However, another study gave the opposite finding: raised diurnal temperature range variation was associated with reduced stroke, especially ischaemic stroke, while raised mean ambient temperature was associated with an increased risk of general stroke, in particular, ischaemic stroke by a magnitude of 7–10%.

The mechanisms for the impact of ambient temperature, including low and high temperatures on stroke risks, morbidity, and mortality, have attracted much literature discussion. Vasoconstriction is postulated to divert blood flow to central organs (brain, heart, and kidneys) in response to a lower ambient temperature, consequently increasing systemic vascular resistance and leading to high blood pressure, which is a known risk factor for stroke (47, 48). It is well established that mean blood pressure tends to be higher in the colder months, and cold exposure can further worsen hypertension in predisposed individuals (49). This exacerbated hypertension may reflect increasing systemic vascular resistance and oxygen demand and ineffective cold adaptation due to autonomic neuropathy in hypertensive patients (50). Another mechanistic explanation indicated that peripheral vasoconstriction may lead to cerebral vasculature congestion, which theoretically could increase the risk of haemorrhage (51). Dehydration is thought to be the potential mechanism for the high ambient temperature associated with increased ischaemic stroke risks, which was demonstrated in a study that dehydration in hospitalised stroke patients is associated with severe stroke morbidity and increased mortality (52).

In reviewing the association between ambient temperature and stroke patients with comorbidity, there were contrasting reported stroke risks. The ischemic stroke patients with comorbidities of hypertension, diabetes, atrial fibrillation, or hyperlipidaemia reported a significant association at a 1°C increase versus an insignificant association at a 5°C increase in mean ambient temperature. However, increased stroke risk was observed with exposure to both low and high ambient temperatures in congestive heart failure patients, as well as exposure to seasonal patterns in patients with hypertension, ischemic stroke, and hypercholesterolemia. In contrast, increased stroke risk was not observed, as compared to summer, spring, autumn, or winter, in stroke patients with comorbidities including hypertension, diabetes, drinking, or smoking.

The mechanism of the contrasted report of significant association in terms of ischaemic stroke risks with comorbidity (hypertension, diabetes, atrial fibrillation, or hyperlipidaemia) at different mean ambient temperatures remains to be established. The apparent observation with 1°C and 5°C increases in mean ambient temperature showed an equivalent baseline mean ambient temperature, and the 1°C increase still made it into the temperature range of below 13^0^C^41^ in contrast to the 5°C increase in the temperature range of well above 13^0^C^34^ (17–19°C), which is a normal range ambient temperature having minimal impacts on stroke risks. The underlying pathology of low and high ambient temperatures increasing the risk of stroke in patients with congestive heart failure included two aspects: (1) low ambient temperature may be mediated by peripheral vasoconstriction and cerebral vasculature congestion (51) due to congestive heart failure; (2) high ambient temperature may be mediated by peripheral vasodilatation, intravascular hypovolemia, and reduced cerebral perfusion due to congestive heart failure. The mechanism of seasonal change having a minimal impact on increasing stroke risk in patients with hypertension, diabetes, drinking, or smoking may be due to a relatively normal ambient temperature (35).

The final discussion point lies in the most recent evidence (53) evaluating the impact of ambient temperature and altitude exposure on stroke burden in a Chinese cohort. This research used data from the National Stroke High-Risk Population Screening System 2020 and assessed the overall prevalence, incidence, and mortality rate of stroke in China by obtaining the annual mean ambient temperature, diurnal temperature range, and altitude for each city. To observe stroke burden through quantifying the three risk elements, including incidence, prevalence, and mortality, the outcome of this research (53) demonstrated a negative linear relationship between mean ambient temperature and stroke risk (incidence and prevalence) as well as a negative association between mean ambient temperature and stroke mortality. A non-linear relationship with decreased risk in both high and low diurnal temperature ranges is observed for stroke burden. Overall, this research outcome (53) is in concert with our systematic review research findings in terms of examining the association between ambient temperature and stroke risk in high-risk populations.

## Limitations

There are three major limitations to this review. First, we have assessed the quality of the included articles but have been unable to incorporate quality scores into the analysis because of the overall poor reporting of results. Second, some of the included studies had small sample sizes, and results were not adjusted for confounders such as air conditioning and heating systems, together with the direct effect of the weather on behaviours, including alcohol intake and activity level, which are crucial confounding factors that are virtually impossible to adjust for in retrospective analyses. Third, we were not able to pool results for the majority of studies due to a lack of crude data and/or inconsistent presentation of data.

## Conclusion

The convincing evidence for an association between ambient temperature and the incidence of stroke lies in the fact that lower temperature was more likely to increase the morbidity and mortality risk of both haemorrhagic and ischaemic stroke in the elderly. In contrast, higher ambient temperature is significantly associated with intracranial haemorrhage risk but decreases risk with ischaemic stroke. Higher and lower ambient temperatures consistently increase stroke risks in patients with comorbidities of congestive heart failure and dyslipidaemia. This evidence implies the need to establish clinical guidelines for preventive intervention in patients with high stroke risks at higher and lower ambient temperatures. Ultimately, further research is needed to explore the relationship between temperature and stroke in different geographical locations, with different assessment tools, such as MRI scans, and with respect to stroke subtypes, including ischaemic and haemorrhagic stroke.

## Data availability statement

All research data are available on reasonable request from the corresponding author.

## Author contributions

ND: Data curation, Formal analysis, Investigation, Methodology, Software, Validation, Visualization, Writing – original draft, Writing – review & editing. CH: Data curation, Formal analysis, Investigation, Methodology, Software, Validation, Visualization, Writing – review & editing. EF: Data curation, Investigation, Methodology, Software, Validation, Visualization, Writing – original draft, Writing – review & editing. JZ: Conceptualization, Supervision, Validation, Visualization, Writing – review & editing. HH: Conceptualization, Supervision, Validation, Visualization, Writing – review & editing. CR: Conceptualization, Resources, Supervision, Validation, Visualization, Writing – review & editing. DX: Conceptualization, Investigation, Methodology, Project administration, Resources, Supervision, Validation, Visualization, Writing – original draft, Writing – review & editing.

## References

[1] Virani SS Alonso A Aparicio HJ Benjamin EJ Bittencourt MS Callaway CW . Heart disease and stroke statistics—2021 update: a report from the American Heart Association. Circulation. (2021) 143:e254–743. doi: 10.1161/CIR.0000000000000950, PMID: 33501848 PMC13036842

[2] Grysiewicz RA Thomas K Pandey DK. Epidemiology of ischemic and hemorrhagic stroke: incidence, prevalence, mortality, and risk factors. Neurol Clin. (2008) 26:871–95. doi: 10.1016/j.ncl.2008.07.003 19026895

[3] Chen J-h Jiang H Wu L Liao X Lu Y Tao X-Q . Association of ischemic and hemorrhagic strokes hospital admission with extreme temperature in Nanchang, China—a case-crossover study. J Clin Neurosci. (2017) 43:89–93. doi: 10.1016/j.jocn.2017.04.044, PMID: 28629681

[4] Gill RS Hambridge HL Schneider EB Hanff T Tamargo RJ Nyquist P. Falling temperature and colder weather are associated with an increased risk of aneurysmal subarachnoid hemorrhage. World Neurosurg. (2013) 79:136–42. doi: 10.1016/j.wneu.2012.06.020, PMID: 22732514

[5] Han J Liu S Zhang J Zhou L Fang Q Zhang J . The impact of temperature extremes on mortality: a time-series study in Jinan, China. BMJ Open. (2017) 7:e014741. doi: 10.1136/bmjopen-2016-014741, PMID: 28465307 PMC5566622

[6] Luo Y Li H Huang F van Halm-Lutterodt N Qin Xu Wang A . The cold effect of ambient temperature on ischemic and hemorrhagic stroke hospital admissions: a large database study in Beijing, China between years 2013 and 2014—utilizing a distributed lag non-linear analysis. Environ Pollut. (2018) 232:90–6. doi: 10.1016/j.envpol.2017.09.021, PMID: 28941717

[7] Yang J Yin P Zhou M Ou CQ Li M Li J . The burden of stroke mortality attributable to cold and hot ambient temperatures: epidemiological evidence from China. Environ Int. (2016) 92-93:232–8. doi: 10.1016/j.envint.2016.04.001 27107228

[8] Zeka A Browne S McAvoy H Goodman P. The association of cold weather and all-cause and cause-specific mortality in the island of Ireland between 1984 and 2007. Environ Health. (2014) 13:1–9. doi: 10.1186/1476-069X-13-104 25480672 PMC4391579

[9] Zheng D Arima H Sato S Gasparrini A Heeley E Delcourt C . Low ambient temperature and intracerebral hemorrhage: the INTERACT2 study. PLoS One. (2016) 11:e0149040. doi: 10.1371/journal.pone.0149040, PMID: 26859491 PMC4747478

[10] Hong Y-C Kim H Oh S-Y Lim Y-H Kim S-Y Yoon H-J . Association of cold ambient temperature and cardiovascular markers. Sci Total Environ. (2012) 435:74–9. doi: 10.1016/j.scitotenv.2012.02.070 22846766

[11] Keatinge W Coleshaw S Cotter F Mattock M Murphy M Chelliah R. Increases in platelet and red cell counts, blood viscosity, and arterial pressure during mild surface cooling: factors in mortality from coronary and cerebral thrombosis in winter. Br Med J. (1984) 289:1405–8. doi: 10.1136/bmj.289.6456.1405, PMID: 6437575 PMC1443679

[12] Lim Y-H Kim H Hong Y-C. Variation in mortality of ischemic and hemorrhagic strokes in relation to high temperature. Int J Biometeorol. (2013) 57:145–53. doi: 10.1007/s00484-012-0542-x, PMID: 22527757

[13] Salam A Kamran S Bibi R Korashy HM Parray A Mannai AA . Meteorological factors and seasonal stroke rates: a four-year comprehensive study. J Stroke Cerebrovasc Dis. (2019) 28:2324–31. doi: 10.1016/j.jstrokecerebrovasdis.2019.05.032, PMID: 31227318

[14] Cowperthwaite MC Burnett MG. An analysis of admissions from 155 United States hospitals to determine the influence of weather on stroke incidence. J Clin Neurosci. (2011) 18:618–23. doi: 10.1016/j.jocn.2010.08.035, PMID: 21398128

[15] Conlon KC Rajkovich NB White-Newsome JL Larsen L O’Neill MS. Preventing cold-related morbidity and mortality in a changing climate. Maturitas. (2011) 69:197–202. doi: 10.1016/j.maturitas.2011.04.004, PMID: 21592693 PMC3119517

[16] Wang X Cao Y Hong D Zheng D Richtering S Sandset E . Ambient temperature and stroke occurrence: a systematic review and meta-analysis. Int J Environ Res Public Health. (2016) 13:698. doi: 10.3390/ijerph13070698, PMID: 27420077 PMC4962239

[17] Lavados PM Olavarría VV Hoffmeister L. Ambient temperature and stroke risk: evidence supporting a short-term effect at a population level from acute environmental exposures. Stroke. (2018) 49:255–61. doi: 10.1161/STROKEAHA.117.017838 29229725

[18] Lian H Ruan Y Liang R Liu X Fan Z. Short-term effect of ambient temperature and the risk of stroke: a systematic review and meta-analysis. Int J Environ Res Public Health. (2015) 12:9068–88. doi: 10.3390/ijerph120809068, PMID: 26264018 PMC4555265

[19] Zorrilla-Vaca A Healy RJ Silva-Medina MM. Revealing the association between cerebrovascular accidents and ambient temperature: a meta-analysis. Int J Biometeorol. (2017) 61:821–32. doi: 10.1007/s00484-016-1260-6, PMID: 27796566

[20] Von Elm E Altman DG Egger M Pocock SJ Gøtzsche PC Vandenbroucke JP. The strengthening the reporting of observational studies in epidemiology (STROBE) statement: guidelines for reporting observational studies. Ann Intern Med. (2007) 147:573–7. doi: 10.7326/0003-4819-147-8-200710160-00010 17938396

[21] Sterne JA Harbord RM. Funnel plots in meta-analysis. Stata J. (2004) 4:127–41. doi: 10.1177/1536867X0400400204

[22] Bai L Li Q Wang J Lavigne E Gasparrini A Copes R . Increased coronary heart disease and stroke hospitalisations from ambient temperatures in Ontario. Heart. (2018) 104:673–9. doi: 10.1136/heartjnl-2017-311821, PMID: 29101264 PMC5890650

[23] Goggins WB Woo J Ho S Chan EY Chau PH. Weather, season, and daily stroke admissions in Hong Kong. Int J Biometeorol. (2012) 56:865–72. doi: 10.1007/s00484-011-0491-9, PMID: 21915799

[24] Guo P Zheng M Wang Y Feng W Wu J Deng C . Effects of ambient temperature on stroke hospital admissions: results from a time-series analysis of 104,432 strokes in Guangzhou, China. Sci Total Environ. (2017) 580:307–15. doi: 10.1016/j.scitotenv.2016.11.093, PMID: 28011022

[25] Hasegawa T Fujikawa M. Environmental factors determine the cerebrovascular disease (CVD) in the Japanese adults. Int Med J. (2015) 22:492–5.

[26] Lee HC Hu CJ Chen CS Lin HC. Seasonal variation in ischemic stroke incidence and association with climate: a six-year population-based study. Chronobiol Int. (2008) 25:938–49. doi: 10.1080/07420520802551469, PMID: 19005897

[27] Lichtman JH Leifheit-Limson EC Jones SB Wang Y Goldstein LB. Average temperature, diurnal temperature variation, and stroke hospitalizations. J Stroke Cerebrovasc Dis. (2016) 25:1489–94. doi: 10.1016/j.jstrokecerebrovasdis.2016.02.037, PMID: 27038980

[28] Lim JS Kwon HM Kim SE Lee J Lee YS Yoon BW. Effects of temperature and pressure on acute stroke incidence assessed using a Korean Nationwide insurance database. J Stroke. (2017) 19:295–303. doi: 10.5853/jos.2017.00045, PMID: 29037003 PMC5647635

[29] Pan WH Li LA Tsai MJ. Temperature extremes and mortality from coronary heart-disease and cerebral infarction in elderly Chinese. Lancet. (1995) 345:353–5. doi: 10.1016/S0140-6736(95)90341-0, PMID: 7845116

[30] Passero S Reale F Ciacci G Zei E. Differing temporal patterns of onset in subgroups of patients with intracerebral hemorrhage. Stroke. (2000) 31:1538–44. doi: 10.1161/01.STR.31.7.1538, PMID: 10884450

[31] Qi XM Wang ZY Xia XS Xue J Gu Y Han S . Potential impacts of meteorological variables on acute lschemic stroke onset. Risk Manag Healthc Policy. (2020) 13:615–21. doi: 10.2147/RMHP.S253559, PMID: 32607029 PMC7311092

[32] Ravljen M Bajrovic F Vavpotic D. A time series analysis of the relationship between ambient temperature and ischaemic stroke in the Ljubljana area: immediate, delayed and cumulative effects. BMC Neurol. (2021) 21:23. doi: 10.1186/s12883-021-02044-8, PMID: 33446129 PMC7807497

[33] Shinkawa A Ueda K Hasuo Y Kiyohara Y Fujishima M. Seasonal-variation in stroke incidence in Hisayama, Japan. Stroke. (1990) 21:1262–7. doi: 10.1161/01.STR.21.9.1262, PMID: 2396260

[34] Toyoda K Koga M Yamagami H Yokota C Sato S Inoue M . Seasonal variations in neurological severity and outcomes of ischemic stroke—5-year single-center observational study—. Circ J. (2018) 82:1443–50. doi: 10.1253/circj.CJ-17-1310, PMID: 29607895

[35] Turin TC Kita Y Murakami Y Rumana N Sugihara H Morita Y . Higher stroke incidence in the spring season regardless of conventional risk factors - Takashima stroke registry, Japan, 1988-2001. Stroke. (2008) 39:745–52. doi: 10.1161/STROKEAHA.107.495929, PMID: 18258821

[36] Vered S Paz S Negev M Tanne D Zucker I Weinstein G. High ambient temperature in summer and risk of stroke or transient ischemic attack: a national study in Israel. Environ Res. (2020) 187:109678. doi: 10.1016/j.envres.2020.109678 32474306

[37] Vodonos A Novack V Horev A Abu Salameh I Lotan Y Ifergane G. Do gender and season modify the triggering effect of ambient temperature on ischemic stroke? Womens Health Issues. (2017) 27:245–51. doi: 10.1016/j.whi.2016.11.002, PMID: 28007390

[38] Wang XY Barnett AG Hu WB Tong SL. Temperature variation and emergency hospital admissions for stroke in Brisbane, Australia, 1996-2005. Int J Biometeorol. (2009) 53:535–41. doi: 10.1007/s00484-009-0241-4, PMID: 19506912

[39] Zheng S Wang MZ Li B Wang S He S Yin L . Gender, age and season as modifiers of the effects of diurnal temperature range on emergency room admissions for cause-specific cardiovascular disease among the elderly in Beijing. Int J Environ Res Public Health. (2016) 13:447. doi: 10.3390/ijerph13050447, PMID: 27128931 PMC4881072

[40] Chang CL Shipley M Marmot M Poulter N. Lower ambient temperature was associated with an increased risk of hospitalization for stroke and acute myocardial infarction in young women. J Clin Epidemiol. (2004) 57:749–57. doi: 10.1016/j.jclinepi.2003.10.016, PMID: 15358404

[41] Palm F Dos Santos M Urbanek C Greulich M Zimmer K Safer A . Stroke seasonality associations with subtype, etiology and laboratory results in the Ludwigshafen stroke study (LuSSt). Eur J Epidemiol. (2013) 28:373–81. doi: 10.1007/s10654-013-9772-4, PMID: 23385658

[42] Ikefuti PV Barrozo LV Braga ALF. Mean air temperature as a risk factor for stroke mortality in São Paulo, Brazil. Int J Biometeorol. (2018) 62:1535–42. doi: 10.1007/s00484-018-1554-y, PMID: 29802502

[43] Fodor DM Fodor M Perju-Dumbrava L. Seasonal variation of stroke occurrence: a hospital based-study. Balneo Res J. (2018) 9:82–7. doi: 10.12680/balneo.2018.178

[44] Oyoshi T Nakayama M Kuratsu J. Relationship between aneurysmal subarachnoid hemorrhage and climatic conditions in the subtropical region, Amami-Oshima, in Japan. Neurol Med Chir (Tokyo). (1999) 39:585–91; discussion 590-581. doi: 10.2176/nmc.39.585, PMID: 10487037

[45] Ranganai E Matizirofa L. An analysis of recent stroke cases in South Africa: trend, seasonality and predictors. S Afr Med J. (2020) 110:92–9. doi: 10.7196/SAMJ.2020.v110i2.013891, PMID: 32657676

[46] Turin TC Kita Y Rumana N Murakami Y Ichikawa M Sugihara H . Stroke case fatality shows seasonal variation regardless of risk factor status in a Japanese population: 15-year results from the Takashima stroke registry. Neuroepidemiology. (2009) 32:53–60. doi: 10.1159/000170907, PMID: 19001797

[47] Woodhouse PR Khaw K-T Plummer M. Seasonal variation of blood pressure and its relationship to ambient temperature in an elderly population. J Hypertens. (1993) 11:1267–74. PMID: 8301109

[48] Ishigami A Hajat S Kovats RS Bisanti L Rognoni M Russo A . An ecological time-series study of heat-related mortality in three European cities. Environ Health. (2008) 7:5. doi: 10.1186/1476-069X-7-5, PMID: 18226218 PMC2266730

[49] Cheng X Su H. Effects of climatic temperature stress on cardiovascular diseases. Eur J Intern Med. (2010) 21:164–7. doi: 10.1016/j.ejim.2010.03.001 20493415

[50] Abboud FM Harwani SC Chapleau MW. Autonomic neural regulation of the immune system: implications for hypertension and cardiovascular disease. Hypertension. (2012) 59:755–62. doi: 10.1161/HYPERTENSIONAHA.111.186833, PMID: 22331383 PMC3313828

[51] McArthur K Dawson J Walters M. What is it with the weather and stroke? Expert Rev Neurother. (2010) 10:243–9. doi: 10.1586/ern.09.154 20136380

[52] Rowat A Graham C Dennis M. Dehydration in hospital-admitted stroke patients detection, frequency, and association. Stroke. (2012) 43:857–9. doi: 10.1161/STROKEAHA.111.640821, PMID: 22156691

[53] Tu WJ Xu Y Fan Y Zeng X Zhao J. Impacts of exposure to ambient temperature and altitude on the burden of stroke. J Neurol. (2023) 270:4214–8. doi: 10.1007/s00415-023-11742-x, PMID: 37166508

